# Kangaroo mother care: a systematic review of barriers and enablers

**DOI:** 10.2471/BLT.15.157818

**Published:** 2015-12-03

**Authors:** Grace J Chan, Amy S Labar, Stephen Wall, Rifat Atun

**Affiliations:** a Department of Global Health and Population, Harvard TH Chan School of Public Health, 677 Huntington Street, Boston, Massachusetts, 02115, United States of America (USA).; b Saving Newborn Lives, Save the Children, Washington, USA.

## Abstract

**Objective:**

To investigate factors influencing the adoption of kangaroo mother care in different contexts.

**Methods:**

We searched PubMed, Embase, Scopus, Web of Science and the World Health Organization’s regional databases, for studies on “kangaroo mother care” or “kangaroo care” or “skin-to-skin care” from 1 January 1960 to 19 August 2015, without language restrictions. We included programmatic reports and hand-searched references of published reviews and articles. Two independent reviewers screened articles and extracted data on carers, health system characteristics and contextual factors. We developed a conceptual model to analyse the integration of kangaroo mother care in health systems.

**Findings:**

We screened 2875 studies and included 112 studies that contained qualitative data on implementation. Kangaroo mother care was applied in different ways in different contexts. The studies show that there are several barriers to implementing kangaroo mother care, including the need for time, social support, medical care and family acceptance. Barriers within health systems included organization, financing and service delivery. In the broad context, cultural norms influenced perceptions and the success of adoption.

**Conclusion:**

Kangaroo mother care is a complex intervention that is behaviour driven and includes multiple elements. Success of implementation requires high user engagement and stakeholder involvement. Future research includes designing and testing models of specific interventions to improve uptake.

## Résumé

**Objectif:**

Étudier les facteurs qui influencent l'adoption de la méthode de la mère «kangourou» dans différents contextes.

**Méthodes:**

Nous avons recherché dans PubMed, Embase, Scopus, Web of Science et les bases de données régionales de l'Organisation mondiale de la Santé des études sur la méthode de la mère «kangourou», les soins «kangourou» ou les soins peau contre peau du 1er janvier 1960 au 19 août 2015, sans restrictions de langues. Nous avons inclus des rapports programmatiques et des références, recherchées manuellement, d'études et d'articles publiés. Deux réviseurs indépendants ont examiné les articles et extrait des données sur les aidants, les caractéristiques des systèmes de santé et les facteurs contextuels. Nous avons élaboré un modèle conceptuel pour analyser l'intégration de la méthode de la mère «kangourou» dans les systèmes de santé.

**Résultats:**

Nous avons examiné 2875 études et inclus 112 études contenant des données qualitatives sur la mise en œuvre. La méthode de la mère «kangourou» a été appliquée de différentes façons selon les contextes. Les études démontrent qu'il existe plusieurs obstacles à la mise en œuvre de la méthode de la mère «kangourou»: elle requiert du temps et un soutien social, et suppose des soins médicaux et une acceptation par les familles. Les obstacles inhérents aux systèmes de santé résidaient notamment dans l'organisation, le financement et la prestation de services. Dans l'ensemble, les normes culturelles ont influencé les perceptions et le succès de l'adoption de cette méthode.

**Conclusion:**

La méthode de la mère «kangourou» est une intervention complexe axée sur le comportement qui inclut de multiples éléments. Le succès de sa mise en œuvre exige un engagement fort des utilisateurs et une mobilisation des parties intéressées. De futurs travaux de recherche incluent la conception et l'essai de modèles d'interventions spécifiques pour favoriser l'adoption de cette méthode.

## Resumen

**Objetivo:**

Investigar los factores que influencian la adopción del método madre canguro en diferentes contextos.

**Métodos:**

Se realizaron búsquedas en las bases de datos PubMed, Embase, Scopus, Web of Science y la Organización Mundial de la Salud sobre estudios relacionados con el “método madre canguro”, “cuidado canguro” o “contacto directo de la piel” desde el 1 de enero de 1960 al 19 de agosto de 2015, sin limitación de idiomas. Se incluyeron informes sistemáticos y búsquedas manuales de referencias de revisiones y artículos publicados. Dos revisores independientes revisaron los artículos y extrajeron datos sobre los cuidadores, las características del sistema sanitario y los factores contextuales. Se desarrolló un modelo conceptual para analizar la integración del método madre canguro en los sistemas sanitarios.

**Resultados:**

Se revisaron 2 875 estudios y se incluyeron 112 estudios que contenían datos cualitativos sobre la implementación. El método madre canguro se aplicó de formas diferentes en contextos diferentes. Los estudios muestran que existen diversas barreras a la hora de implementar el método madre canguro, incluyendo la necesidad de tiempo, apoyo social, asistencia médica y aceptación familiar. Las barreras dentro de los sistemas sanitarios incluían la organización, la financiación y el suministro de servicios. En el contexto general, las normas culturales influenciaron las percepciones y el éxito de la adopción.

**Conclusión:**

El método madre canguro es una intervención compleja impulsada por el comportamiento e incluye múltiples elementos. El éxito de la implementación requiere una participación elevada del usuario y la involucración del interesado. Las futuras investigaciones incluyen diseñar y probar modelos de intervenciones específicas para mejorar la aceptación.

## ملخص

**الهدف:**

النظر في العوامل التي تؤثر على تبني طريقة رعاية الأمهات لمواليدهن على طريقة الكنغر في سياقات مختلفة.

**الطريقة:**

لقد بحثنا في قواعد بيانات PubMed و Embase و Scopus و Web of Science ****وقواعد البيانات الإقليمية التابعة لمنظمة الصحة العالمية لإيجاد دراسات حول "رعاية الأم لوليدها على طريقة الكنغر" أو "الرعاية على طريقة الكنغر" أو "رعاية الوليد بملامسة بشرة الأم" بدءًا من 1 يناير/كانون الثاني 1960 وحتى 19 أغسطس/آب 2015 من دون القيود اللغوية. وقمنا بتضمين التقارير الصادرة عن البرامج ومراجع المقالات والمراجعات التي تم إجراء البحث بشأنها بشكل يدوي. ثم قام اثنان من المراجعين المستقلين بتصفح المقالات واستخلاص البيانات حول مقدمي الرعاية، وخصائص النظام الصحي، والعوامل السياقية. كما قمنا بوضع نموذج تصوري لتحليل عملية إدماج رعاية الأم لوليدها على طريقة الكنغر في الأنظمة الصحية.

**النتائج:**

قمنا بفحص دراسات بلغ عددها 2875 دراسة، كما قمنا بتضمين 112 دراسة اشتملت على بيانات نوعية حول التنفيذ. كانت رعاية الأم لوليدها على طريقة الكنغر قد تم تطبيقها بطرق مختلفة في سياقات مختلفة. وتكشف الدراسات عن وجود العديد من العوائق في طريق تطبيق رعاية الأم لوليدها على طريقة الكنغر، من بينها الحاجة إلى الوقت، والدعم الاجتماعي، والرعاية الصحية، وتقبل الرعاية من جانب الأسرة. ومن بين العوائق الكامنة في النظم الصحية كانت هناك النواحي التنظيمية، والتمويل، وتقديم الخدمة. وفي السياق الأعم، أثرت المعايير الثقافية على التصورات المحيطة بهذا النوع من الرعاية ومدى نجاح تطبيقه.

**الاستنتاج:**

تمثل رعاية الأم لوليدها على طريقة الكنغر وسيلة معقدة للتدخل تعتمد على السلوك، وتتضمن عدة عناصر. ويحتاج نجاح تطبيقها إلى مشاركة كبيرة من جانب المستفيدين فضلاً عن إدراج جهود الجهات المعنية. وتتضمن البحوث المستقبلية نماذج لتصميم واختبار عمليات تدخل محددة بغرض زيادة تبني هذا النوع من الرعاية.

## 摘要

**目的:**

旨在调查不同环境下采用袋鼠妈妈式护理的影响因素。

**方法:**

我们搜索了 PubMed、Embase、Scopus、Web of Science 以及世界卫生组织的区域数据库，以查找 1960 年 1 月 1 日到 2015 年 8 月 19 日期间关于“袋鼠妈妈式护理”或“袋鼠式护理”或“肌肤接触护理”的研究。我们包括了项目报告以及手工检索到已发表的评论与文章等参考资料。两个独立的评论员分别筛选出关于护理人员、卫生系统特征和环境因素的文章并提取了数据。我们开发了一个概念模型，以分析袋鼠妈妈式护理在卫生系统中的整合。

**结果:**

我们筛选出 2875 项研究，其中 112 项研究中包含实施方面的定性数据。 袋鼠妈妈式护理以各种方式应用于不同的环境中**。**该研究表明实施袋鼠妈妈式护理存在几个障碍，包括需要时间、社会支持、医疗护理和家庭的接受。卫生系统内部的障碍包括组织、筹资和提供服务。在大环境中，文化规范影响人们对采用该护理方式的看法和成功率。

**结论:**

袋鼠妈妈式护理是一种复杂的干预措施，受行为驱动且涵盖多种因素。 成功的实施要求用户的高度参与以及其他利益相关者的参与。未来的研究包括具体干预措施的设计和测试模型，以提高该护理方式的接受率。

## Резюме

**Цель:**

Изучить факторы, влияющие на применение метода «кенгуру» в различных контекстах.

**Методы:**

Нами был проведен поиск по базам данных PubMed, Embase, Scopus, Web of Science, а также по региональным базам данных Всемирной организации здравоохранения; целью поиска были исследования по теме «метод "кенгуру"», или «принцип "кенгуру"», или «метод телесного контакта» в период с 1 января 1960 г. по 19 августа 2015 г. без ограничений по языку. Мы включили в рассмотрение отчеты о программах и найденные вручную ссылки на опубликованные обзоры и статьи. Два независимых эксперта просматривали статьи и извлекали из них данные о лицах, осуществляющих уход, о характеристиках систем здравоохранения и факторах, определяющих контекст. Мы разработали концептуальную модель для анализа интеграции метода «кенгуру» в системы здравоохранения.

**Результаты:**

Мы проверили 2875 исследований и включили в обзор 112 исследований, которые содержали количественные данные, касающиеся осуществления метода. В различных контекстах способы осуществления ухода по методу «кенгуру» были различными. Исследования показали, что существует несколько препятствий на пути осуществления ухода методом «кенгуру», включая потребность во времени, социальной поддержке, медицинском уходе, а также в принятии со стороны семьи. Препятствия со стороны системы здравоохранения включают организационные аспекты, финансирование и предоставление услуг. В широком контексте культурные нормы оказывали влияние на восприятие и успех осуществления метода.

**Вывод:**

Уход по методу «кенгуру» представляет собой комплексное вмешательство, в основе которого лежат поведенческие факторы и которое включает множество элементов. Для успешного осуществления необходима высокая степень заинтересованности участников, а также привлечение партнеров. В будущих исследованиях планируется уделить время разработке и тестированию моделей конкретных вмешательств с целью улучшения усвоения метода.

## Introduction

More than 2.7 million newborns die each year, accounting for 44% of children dying before the age of five years worldwide. Complications of preterm birth are the leading cause of death among newborns.^1^ Kangaroo mother care can include early and continuous skin-to-skin contact, breastfeeding, early discharge from the health-care facility and supportive care.^2^ The clinical efficacy and health benefits of kangaroo mother care have been demonstrated in multiple settings. In low birthweight newborns (< 2000 g) who are clinically stable, kangaroo mother care reduces mortality and if widely applied could reduce deaths in preterm newborns.^3^^,^^4^ However, in spite of the evidence, country-level adoption and implementation of kangaroo mother care has been limited and global coverage remains low. Few studies have examined the reasons for the poor uptake of kangaroo mother care.

To understand factors influencing adoption of kangaroo mother care in different contexts, we did a systematic review. We created a narrative analysis of the articles and reports identified, guided by a conceptual framework^5^ with five elements: (i) the problem being addressed – neonatal mortality; (ii) the intervention or innovation aimed at addressing the problem; (iii) the adoption system – those implementing the intervention, those benefiting from it and those affected by it; (iv) the health system – organization, financing and service delivery; and (v) the broad context – demographic, epidemiological, political, economic and sociocultural factors. These five elements interact to influence the extent, pattern and rate of adoption of interventions in health systems.^5^

## Methods

We searched PubMed, Embase, Web of Science, Scopus, African Index Medicus (AIM), Latin American and Caribbean Health Sciences Literature (LILACS), Index Medicus for the Eastern Mediterranean Region (IMEMR), Index Medicus for the South-East Asian Region (IMSEAR) and Western Pacific Region Index Medicus (WPRIM) without language restrictions, from 1 January 1960 to 19 August 2015 using the search terms “kangaroo mother care” or “kangaroo care” or “skin-to-skin care.” We excluded studies without human subjects or without primary data collection. We screened studies for inclusion if they discussed barriers to kangaroo mother care implementation or enablers for successful implementation. Our population of interest included mothers, newborns or mother-newborn dyads who had practiced kangaroo mother care, and health-care providers, health facilities, communities and health systems that have implemented such care. We hand-searched the reference lists of published systematic reviews and references of the included articles. To search the grey literature for unpublished studies, we explored programmatic reports and requested data from programmes implementing kangaroo mother care.

Two reviewers independently extracted data from identified articles using standardized forms to identify potential determinants of kangaroo mother care uptake, including data on knowledge, attitudes and practices. Reviewers compared their results to reach consensus and ties were broken by a third party. To assess study quality, we evaluated each study in five quality domains: selection bias, appropriateness of data collection, appropriateness of data analysis, generalizability and ethical considerations.^6^

A deductive approach was used to fit the outputs of the analysis to the elements of the conceptual framework and explore emerging themes.^7^ Using the qualitative analytical software NVivo (QSR International, Melbourne, Australia), two researchers indexed and annotated the data through several rounds of coding to analyse themes, viewpoints, ideas and experiences. Once major themes were established, we constructed narratives and categorized the data into matrices by theme. We highlighted quotes that summarized multiple perspectives from the articles. Narratives and matrices were used to define specific concepts and explore associations between themes.

Themes were explored at each level of implementation (mothers, fathers and families; health-care workers; facilities). We examined the interactions between implementers and described health system characteristics that could influence the uptake of kangaroo mother care.

## Results

**Fig. 1:**
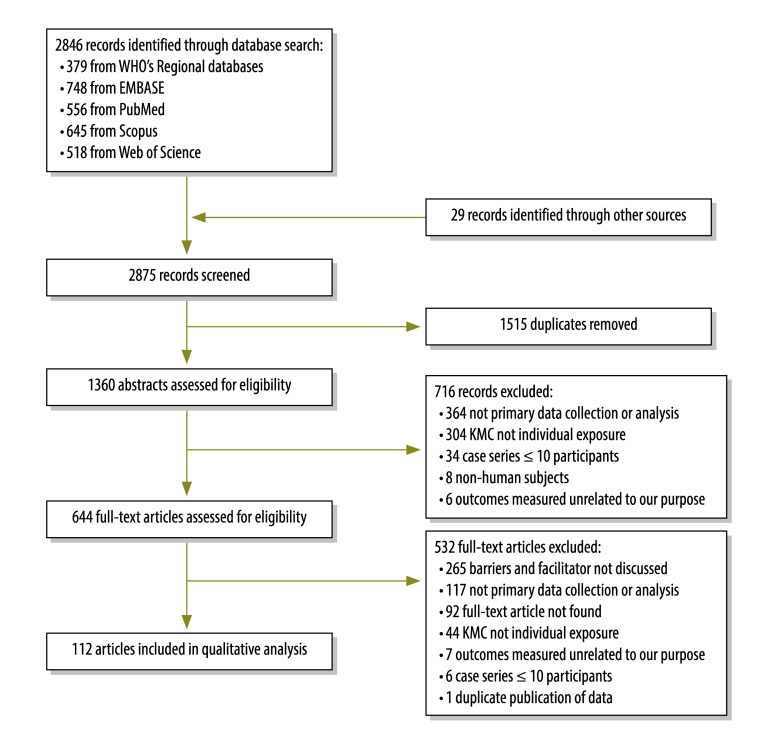
Flowchart showing the selection of studies on kangaroo mother care (KMC)

Of the 2875 papers identified, we included 112 studies with qualitative data on barriers to and enablers of kangaroo mother care (Fig. 1). Most of the studies were published between 2010 and 2015 (66; 59%) and had less than 50 participants (67; 60%). Nearly half of the studies were surveys or interviews (50; 45%). Forty studies (36%) were conducted in the WHO Region of the Americas; 29 (26%) in WHO African Region; 64 (57%) in countries with low neonatal mortality, defined as less than 15 deaths per 1000 live births;^8^ 48 (43%) in urban settings; and 67 (60%) at health facilities. Many studies did not include neonatal characteristics such as gestational age (68; 61%) or weight (75; 67%; Table 1). The majority (68; 60%) of the studies appropriately addressed at least four of the five quality domains.

**Table 1:** Characteristics of included studies in the systematic review on kangaroo mother care

Study characteristic	No. (%) of studies (*n* = 112)
**Year**	
2015^9^^–^^15^	7 (6)
2010 to 2014^16^^–^^75^	59 (53)
2000 to 2009^76^^–^^115^	40 (36)
1988 to 1999^116^^–^^120^	5 (5)
**No. of participants**	
< 50^10^^–^^12^^,^^14^^,^^15^^,^^17^^,^^22^^,^^24^^–^^26^^,^^28^^–^^31^^,^^33^^,^^35^^,^^36^^,^^39^^–^^41^^,^^45^^,^^47^^,^^50^^,^^52^^,^^53^^,^^55^^–^^57^^,^^59^^,^^60^^,^^63^^,^^64^^,^^67^^,^^69^^,^^72^^,^^74^^,^^77^^,^^79^^,^^80^^,^^83^^–^^87^^,^^89^^–^^97^^,^^99^^–^^103^^,^^106^^,^^108^^,^^110^^–^^112^^,^^114^^,^^115^^,^^117^	66 (59)
50 to < 100^13^^,^^16^^,^^20^^,^^21^^,^^27^^,^^32^^,^^37^^,^^42^^–^^44^^,^^51^^,^^66^^,^^68^^,^^71^^,^^118^^,^^120^	15 (13)
100 to < 200^23^^,^^46^^,^^48^^,^^54^^,^^61^^,^^65^^,^^73^^,^^78^^,^^82^^,^^88^^,^^104^^,^^105^^,^^107^^,^^109^	15 (13)
≥ 200^9^^,^^18^^,^^19^^,^^34^^,^^38^^,^^49^^,^^58^^,^^62^^,^^70^^,^^75^^,^^76^^,^^81^^,^^98^^,^^113^^,^^116^^,^^119^	16 (14)
**Study type**	
Survey or interview^11^^–^^14^^,^^16^^,^^18^^,^^21^^,^^28^^,^^29^^,^^32^^,^^33^^,^^35^^,^^39^^–^^45^^,^^48^^–^^52^^,^^58^^,^^63^^,^^64^^,^^66^^,^^69^^,^^72^^,^^74^^,^^75^^,^^77^^,^^79^^,^^87^^,^^89^^–^^91^^,^^94^^–^^97^^,^^101^^,^^102^^,^^106^^,^^107^^,^^111^^,^^114^^,^^115^^,^^117^	50 (45)
Facilities’ evaluation^24^^,^^25^^,^^27^^,^^31^^,^^34^^,^^47^^,^^53^^–^^55^^,^^57^^,^^59^^,^^60^^,^^67^^,^^80^^,^^82^^,^^83^^,^^100^^,^^108^^,^^113^	19 (17)
Randomized control trial^9^^,^^10^^,^^37^^,^^61^^,^^68^^,^^76^^,^^99^^,^^103^^,^^105^^,^^110^^,^^112^^,^^119^	12 (11)
Cohort study^23^^,^^56^^,^^81^^,^^92^^,^^116^	5 (4)
Other (chart review, case control, surveillance)^15^^,^^17^^,^^19^^,^^20^^,^^22^^,^^26^^,^^30^^,^^36^^,^^38^^,^^46^^,^^62^^,^^65^^,^^70^^,^^71^^,^^78^^,^^84^^–^^86^^,^^88^^,^^98^^,^^104^^,^^109^^,^^118^^,^^120^	24 (21)
Pre-post^73^	1 (1)
Interventional trial^93^	1 (1)
**WHO region**	
Americas^12^^,^^21^^,^^28^^,^^33^^–^^37^^,^^42^^–^^44^^,^^50^^,^^52^^,^^56^^,^^63^^,^^65^^,^^71^^–^^75^^,^^84^^–^^91^^,^^94^^,^^97^^,^^101^^,^^106^^,^^108^^,^^112^^–^^115^^,^^119^^,^^120^	40 (36)
African^9^^–^^11^^,^^16^^,^^17^^,^^20^^,^^23^^–^^26^^,^^29^^,^^47^^,^^51^^,^^55^^,^^58^^–^^60^^,^^68^^,^^80^^–^^83^^,^^92^^,^^96^^,^^99^^,^^100^^,^^102^^,^^110^^,^^116^	29 (26)
European^13^^–^^15^^,^^38^^–^^41^^,^^45^^,^^48^^,^^49^^,^^53^^,^^54^^,^^64^^,^^66^^,^^70^^,^^95^^,^^104^^,^^107^^,^^118^	19 (17)
South-East Asia^18^^,^^19^^,^^22^^,^^30^^,^^32^^,^^67^^,^^76^^,^^77^^,^^93^^,^^98^^,^^103^^,^^109^	12 (11)
Eastern Mediterranean^46^^,^^61^^,^^62^^,^^69^	4 (3)
Western Pacific^31^^,^^78^^,^^105^^,^^111^	4 (3)
Multiple regions^27^^,^^57^^,^^79^	3 (3)
Missing^117^	1 (1)
**Country-level neonatal mortality rate (deaths per 1000 live birth)**	
< 5^14^^,^^15^^,^^36^^–^^45^^,^^48^^,^^49^^,^^52^^–^^54^^,^^56^^,^^63^^–^^66^^,^^70^^,^^71^^,^^82^^,^^94^^,^^95^^,^^104^^–^^108^^,^^111^^–^^113^^,^^120^	36 (32)
5 to < 15^12^^,^^21^^,^^28^^,^^33^^–^^35^^,^^46^^,^^50^^,^^58^^,^^59^^,^^61^^,^^62^^,^^69^^,^^74^^,^^75^^,^^84^^–^^91^^,^^97^^,^^101^^,^^114^^,^^115^^,^^119^	28 (25)
15 to < 30^9^^–^^11^^,^^16^^–^^19^^,^^22^^–^^26^^,^^29^^,^^30^^,^^32^^,^^47^^,^^51^^,^^57^^–^^60^^,^^68^^,^^76^^–^^78^^,^^80^^–^^83^^,^^93^^,^^98^^–^^100^^,^^102^^,^^103^^,^^109^^,^^110^	37 (33)
≥ 30 ^50^^, ^^57^^, ^^88^	4 (4)
Missing^13^^,^^20^^,^^27^^,^^31^^,^^73^^,^^79^^,^^117^	7 (6)
**Setting**	
Urban^17^^,^^23^^,^^28^^,^^33^^,^^35^^,^^36^^,^^38^^,^^39^^,^^41^^,^^43^^,^^44^^,^^49^^,^^50^^,^^52^^,^^56^^,^^60^^,^^61^^,^^63^^,^^65^^–^^67^^,^^72^^,^^77^^,^^78^^,^^80^^,^^81^^,^^87^^,^^89^^–^^92^^,^^96^^,^^97^^,^^100^^–^^102^^,^^105^^,^^106^^,^^108^^,^^109^^,^^111^^,^^114^^–^^120^	48 (43)
Urban and rural^19^^,^^34^^,^^42^^,^^58^^,^^62^^,^^70^^,^^75^^,^^79^^,^^84^^,^^85^^,^^88^^,^^99^^,^^104^^,^^110^^,^^113^	15 (13)
Rural^16^^,^^21^^,^^51^^,^^68^^,^^76^^,^^98^	6 (5)
Missing^9^^–^^15^^,^^18^^,^^20^^,^^22^^,^^24^^–^^27^^,^^29^^–^^32^^,^^37^^,^^40^^,^^45^^–^^48^^,^^53^^–^^55^^,^^57^^,^^59^^,^^64^^,^^69^^,^^71^^,^^73^^,^^74^^,^^82^^,^^83^^,^^86^^,^^93^^–^^95^^,^^103^^,^^107^^,^^112^	43 (38)
**Population source**	
Health facility^10^^,^^11^^,^^13^^,^^14^^,^^16^^,^^17^^,^^23^^–^^30^^,^^33^^–^^36^^,^^41^^,^^46^^,^^47^^,^^49^^,^^50^^,^^52^^,^^55^^–^^57^^,^^59^^–^^61^^,^^64^^,^^67^^,^^69^^–^^71^^,^^75^^,^^76^^,^^78^^–^^92^^,^^94^^,^^96^^,^^97^^,^^99^^,^^100^^,^^102^^,^^106^^,^^108^^,^^110^^,^^113^^–^^116^^,^^118^^,^^119^	67 (60)
Neonatal intensive care unit or stepdown unit^12^^,^^15^^,^^22^^,^^31^^,^^37^^–^^40^^,^^42^^–^^45^^,^^48^^,^^53^^,^^54^^,^^63^^,^^65^^,^^66^^,^^72^^–^^74^^,^^93^^,^^95^^,^^103^^–^^105^^,^^107^^,^^109^^,^^111^^,^^112^^,^^117^^,^^120^	32 (28)
Community or population-based surveillance^9^^,^^18^^,^^19^^,^^21^^,^^32^^,^^51^^,^^58^^,^^62^^,^^68^^,^^77^^,^^98^^,^^101^	12 (11)
Missing^20^	1 (1)
**Gestational age**	
Preterm 34 to < 37 weeks^15^^,^^16^^,^^35^^,^^50^^,^^72^^,^^84^^,^^87^^,^^97^^,^^102^^,^^114^^,^^117^^,^^118^^,^^120^	13 (12)
All gestational ages^9^^,^^10^^,^^19^^,^^36^^,^^38^^,^^39^^,^^58^^,^^62^^,^^68^^,^^76^^,^^77^^,^^98^	12 (11)
Very preterm < 34 weeks^40^^,^^48^^,^^63^^–^^65^^,^^70^^,^^95^^,^^101^^,^^112^	9 (8)
Mixed preterm and very preterm < 37 weeks^33^^,^^37^^,^^89^^,^^90^^,^^94^^,^^109^	6 (5)
Full term ≥ 37 weeks^41^^,^^49^^,^^61^^,^^71^	4 (3)
Missing^11^^–^^14^^,^^17^^,^^18^^,^^20^^–^^32^^,^^34^^,^^42^^–^^47^^,^^51^^–^^57^^,^^59^^,^^60^^,^^66^^,^^67^^,^^69^^,^^73^^–^^75^^,^^78^^–^^83^^,^^85^^,^^86^^,^^88^^,^^91^^–^^93^^,^^96^^,^^99^^,^^100^^,^^103^^–^^108^^,^^110^^,^^111^^,^^113^^,^^115^^,^^116^^,^^119^	68 (61)
**Birthweight**	
Low birthweight 1500 to < 2500 g^33^^,^^50^^,^^51^^,^^72^^,^^80^^,^^81^^,^^85^^,^^88^^,^^91^^,^^93^^,^^96^^,^^116^^,^^119^	13 (12)
All birthweights^9^^,^^10^^,^^19^^,^^36^^,^^38^^,^^39^^,^^48^^,^^58^^,^^62^^,^^68^^,^^76^^,^^77^^,^^98^	13 (12)
Mixed low and very low birthweight < 2500 g^17^^,^^23^^,^^90^^,^^92^^,^^101^^,^^109^^,^^120^	7 (6)
Very low birthweight < 1500 g^78^^,^^89^^,^^103^^,^^105^	4 (3)
Missing^11^^–^^16^^,^^18^^,^^20^^–^^22^^,^^24^^–^^32^^,^^34^^,^^35^^,^^37^^,^^40^^–^^47^^,^^49^^,^^52^^–^^57^^,^^59^^–^^61^^,^^63^^–^^67^^,^^69^^–^^71^^,^^73^^–^^75^^,^^79^^,^^82^^–^^84^^,^^86^^,^^87^^,^^94^^,^^95^^,^^97^^,^^99^^,^^100^^,^^102^^,^^104^^,^^106^^–^^108^^,^^110^^–^^115^^,^^117^^,^^118^	75 (67)

WHO: World Health Organization.

Note: Inconsistencies arise in some values due to rounding.

### Conceptual framework

#### Problem

The narrative synthesis of the studies showed that the burden of death and disability of newborns was acknowledged as an important problem.^9^^–^^11^^,^^16^^–^^32^^,^^76^^–^^83^

#### Intervention

The included studies revealed that kangaroo mother care is a complex intervention with several possible components – skin-to-skin contact, breastfeeding, early discharge and follow-up (Table 2). The included components varied across locations and by individual implementer.

**Table 2:** Descriptions of kangaroo mother care in studies included in the systematic review

Characteristic	Common theme	Less common theme	Quotation
Duration skin-to-skin contact	As long as possible 24 hours/day Early/prolonged/continuous 2 hours or more per day To begin once newborn had stabilized	During breastfeeding Less than 24 hours/day To begin immediately after birth To begin 24 hours after birth	“Kangaroo mother care is defined as early, prolonged and continuous (or as far as circumstances permit) skin-to-skin care between the low birthweight infant and mother.”^39^
Extended duration skin-to-skin contact	As long as possible As long as circumstances permit Until newborn weight of 2500 g	First month of life Until 24 hours after birth Until 37 weeks post menstrual age	“Mothers were instructed to continue kangaroo position at least until the baby reached 2500 g.”^116^
Breastfeeding	Exclusive On demand Breastfeeding encouraged Breastfeeding would begin only after skin-to-skin contact had been completed for a given period of time	Kangaroo mother care integrated as part of a larger breastfeeding package Discharge after breastfeeding established Breastfeeding only after suturing and skin-to-skin contact had been completed	“Exclusive breastfeeding wherever possible and early discharge from the health facility when breastfeeding has been established.”^88^
Newborn clothing	Blanket cover Naked Diaper	Cap Booties	“Undressed except for a diaper and was covered with the mother’s gown and a baby sheet.”^93^
Newborn position	Sleeping upright Vertical against chest Between mother’s breasts skin-to-skin contact Held after being removed from incubator Prone	Upright On adult’s chest On mother’s or father’s chest Vertical under clothes Prone position Against mother’s chest	“The baby is kept upright, close to the chest of the adult.”^84^
Bathing	Clean baby with damp or dry cloth	Dry infant after birth	“The routines included quickly drying the newborn immediately after birth and then placing it naked (skin-to-skin) on the mother’s chest.”^41^
Caregiver clothing	Open gown Wrap (cloth or blanket)	Dupatta Specialized kangaroo mother care bra	“Held in position by using innovations like dupatta (stole), sports bra, loose blouse or a specially designed sling.”^109^
Caregiver position	Upright Prone Inclined	Seated in chair Walking around	“Skin-to-skin contact prone or semi-upright position.”^101^
Early discharge	Early discharge (undefined) Early discharge based on clinical conditions Infant weight gain, mother competency in kangaroo mother care	Skin-to-skin contact encouraged before discharge Discharge after breastfeeding established	“Discharge when the mother shows an appropriate level of infant-handling competency and the infant is gaining weight.”^33^
Follow-up	Follow up (undefined) Adequate follow-up Within the facility at: 1−2 weeks 1–6 months 1 year	As part of Brazilian Ministry of Health guidelines: Week 1: 3 times (home) Week 2: 2 times (home) Week 3: 1 time (home)	“With a proper follow-up system in place for regular review of the infant.”^90^

Note: The quotes were concise examples of common themes found across many articles.

The promotion of skin-to-skin contact for as long as possible once the newborn was stabilized emerged as a common theme in several studies.^33^^–^^35^^,^^84^^–^^91^^,^^116^ However, there was limited information on the recommended frequency and duration of skin-to-skin contact and the specific criteria for stopping skin-to-skin contact.^31^^,^^36^^–^^38^^,^^89^^,^^92^^,^^93^^,^^117^

#### Implementation

The complexity of kangaroo mother care and lack of a standardized operational definition makes it challenging to implement. Implementation of kangaroo mother care can be considered at three levels: (i) mothers, fathers and families; (ii) health-care workers; and (iii) facilities. The location of facilities and the resources available determine whether kangaroo mother care takes place in the health facility or at home.^18^^,^^27^^,^^33^

Mothers, fathers and families were usually the primary caregivers of preterm newborns and involved in decision-making and practice of care.^11^^,^^16^^,^^94^^,^^95^^,^^117^ Health-care workers were critical for implementation in hospitals or health facilities. Their main role was to educate the parents about kangaroo mother care.

We identified six major themes concerning barriers and enablers for implementation of kangaroo mother care: (i) buy-in and bonding; (ii) social support; (iii) time; (iv) medical concerns; (v) access and (vi) context (Table 3).

**Table 3:** Summary of enablers and barriers to implementation of kangaroo mother care

Level of implementation	Adoption systems				Health systems access	Context, cultural norms
	Buy-in and bonding	Social support	Access	Medical concerns		
**Parents**						
Enablers	Calming, natural, instinctive, healing for parents and infant	Father, health-care worker, family and community support for mothers and fathers was crucial to success of kangaroo mother care	Kangaroo mother care at home allowed parents to perform other duties	Helped mothers recover emotionally	Belief that kangaroo mother care was cheaper than incubator care	Mother preferred kangaroo mother care to incubator, inspired confidence Gender equality
Barriers	Stigma, shame, kangaroo mother care felt forced	Fear, guilt, discomfort of family members to participate or condone kangaroo mother care in public Privacy	Caregivers were unable to devote time Mothers lonely in kangaroo mother care ward	Maternal fatigue and pain	Associated costs Transport	Traditional, bathing, carrying and breastfeeding practices did not always align with kangaroo mother care guidelines
**Health-care workers**						
Enablers	Nurses more likely to use kangaroo mother care after seeing positive effects. Support from more experienced nurses improved buy-in	Management promotion of kangaroo mother care Role of parents and other health-care workers	Kangaroo mother care did not increase workload	Temperature stability. Experienced nurses more comfortable with kangaroo mother care	Virtual communication and training. Integration of kangaroo mother care into health-care curriculum	None
Barriers	Nurses fail to have strong belief in importance of kangaroo mother care Inconsistent knowledge and application of kangaroo mother care	Management did not prioritize kangaroo mother care Parents could serve as a hindrance to health-care worker	Extra workload Takes away time from other patients	Nurses did not feel kangaroo mother care appropriate for infants who they felt were too small/young/ill	Difficulty finding time for training Inadequate/inconsistent training	Traditional protocols interfered (bathing, carrying) Nurse excluding father from infant care was a cultural norm
**Facilities**						
Enablers	*Leadership* Management support	*Staffing support* Good communication Use of committees to advocate for kangaroo mother care	Unlimited visitation preferred	Access to private space including family rooms or privacy screen. Higher breast milk feeding rates at discharge when breast feeding was allowed and encouraged throughout the hospital	*Access to structural resources* Quiet atmosphere within facilities allows mothers to rest Breast milk banks provide milk and can be an educational tool among mothers	*Reporting and data* Collection of data Use of performance standards and quality improvement measures Site assessment tools
Barriers	Leadership lack of buy-in led to lack of adequate resources	Staffing shortages, high staff and leadership turnover Staff resisted changing protocols	There was limited visitation time due to staff shortages	Disagreement over clinical stability Facilities did not provide food for mothers Only low birthweight infants received kangaroo mother care in some locations	Lack of money at the facility for mother’s transportation Distance to the hospital for mothers without hospital-provided transportation Lack of space and privacy for mothers to do kangaroo mother care Lack of money for transportation, beds and kangaroo mother care wrappers Poor management of resources donated to the hospital	Lack of use of data to document skin-to-skin contact practised on electronic medical record Nurses not given feedback on kangaroo mother care data collected Visitation policies sometimes prevented mothers from performing skin-to-skin contact continuously. Staff found visitors get in the way.

#### Buy-in and bonding

Buy-in and bonding refer to the acceptance of kangaroo mother care, belief in the benefits of such care to mothers and preterm or low birthweight infants and reported perceptions of bonding. Fear, stigma and/or anxiety about having a preterm infant impaired the care process. Mothers felt shame or guilt for having a preterm infant^96^^,^^97^ and some did not want to keep their baby.^16^

Positive perceptions of the potential benefits of kangaroo mother care for caregivers and for newborns among mothers, fathers and families promoted uptake. Studies used words such as relaxed, calm, happy, natural, instinctive and safe to describe the bonding process that mothers and fathers reported during and after kangaroo mother care.^35^^,^^39^^,^^40^^,^^94^^,^^95^^,^^98^ Mothers observed their newborns sleeping longer during skin-to-skin contact; infants were described as less anxious, more restful, more willing to breastfeed and happier than when in an incubator.^41^^,^^121^

A lack of belief in kangaroo mother care and limited knowledge of such care restricted its uptake among health-care workers.^39^^,^^42^^–^^45^ In some facilities, there was reluctance by management to allocate dedicated space to kangaroo mother care or to rearrange staffing schedules to allow for supervision of kangaroo mother care.^12^^,^^16^^,^^22^^,^^25^^,^^29^^,^^36^^,^^46^^,^^82^^,^^99^^,^^122^ Facility leadership had high turnover as leaders trained in kangaroo mother care frequently left for better positions.^25^^,^^27^^,^^29^^,^^42^^,^^47^^,^^82^^,^^99^^,^^100^^,^^123^ On the other hand, facilities that had successfully implemented kangaroo mother care reported support from management and good communication among the staff.^24^^,^^42^

#### Social support

Social support refers to assistance received from other people to perform kangaroo mother care. While practicing kangaroo mother care, both mothers and fathers did not feel supported by their families or communities.^35^^,^^96^ Mothers experienced a lack of support from health-care workers. In settings like Zimbabwe, fathers voiced unease about performing kangaroo mother care because of societal norms that childcare should be the role of the mother.^79^^,^^96^ In contrast, among mothers, fathers and families, uptake was promoted by societal acceptance of paternal participation in childcare, by family and community acceptance of kangaroo mother care and by the presence of engaged health-care workers.^32^^,^^48^ In societies where gender roles were more equal (e.g. Scandinavian countries), there were fewer barriers to fathers performing kangaroo mother care.^48^^,^^49^ Paternal involvement played a large role in uptake – either by division of labour or by helping the mother feel comfortable. In Brazil, mothers were grateful to have someone help them during kangaroo mother care, such as grandmothers and sisters, who could take care of housework and help with the newborn.^101^ Within the maternity ward, peer support from other mothers through sharing their kangaroo mother care experiences also helped promote acceptance.^79^^,^^102^

When institutional leadership did not prioritize kangaroo mother care, health-care workers were less motivated to practice or teach it,^42^^,^^44^ but felt empowered to do so when management allowed for roles in decision-making, promoted kangaroo mother care or mobilized resources for it.^24^ Staffing shortages and staff turnover created barriers to implementation of kangaroo mother care within a facility.^42^ By contrast, effective coordination of and communication between staff helped facilitate implementation.^82^

#### Time

The time needed to provide kangaroo mother care was a potential barrier for mothers, fathers and families, due to responsibilities at home and work and time needed for commuting, preventing them from devoting the time needed for continuous and extended kangaroo mother care.^16^^,^^39^^,^^41^^,^^50^^,^^79^^,^^91^^,^^102^ Conversely, practice of such care at home promoted its uptake.^92^ High workload of health-care workers did not allow sufficient time to dedicate to teaching kangaroo mother care, which further increased workload, especially in facilities with staffing shortages.^78^^,^^79^^,^^103^

One study showed that uptake of kangaroo mother care increased with expansion of visiting hours at health facilities.^104^

#### Medical concerns

Clinical conditions of the mother and/or newborn may prevent kangaroo mother care from occurring. The medical effects of delivery for mothers, including fatigue, depression and postpartum pain, especially after a caesarean section, can reduce uptake of kangaroo mother care.^48^^,^^51^^,^^52^^,^^77^^,^^98^ Particularly for very preterm or unstable infants, concern about potential adverse consequences, such as fear of dislocation of intravenous lines, was an obstacle to kangaroo mother care.^38^^,^^53^^,^^54^ Knowledge that kangaroo mother care supported newborns in stabilizing their temperatures, helped with breathing and promoted mother–child bonding, encouraged its use.^118^

#### Access

While parents believed that kangaroo mother care was less costly than incubator care,^96^ lack of money for transportation and the distance to hospital were often reported as the biggest challenges^55^^,^^81^^,^^82^^,^^105^ as were low resources for newborn-care services.^82^ Lack of private space for mothers to perform kangaroo mother care and to remain in the hospital with the newborn hindered its uptake,^24^^,^^25^ as did allocation of resources intended for kangaroo mother care to other programmes.^24^ Uptake improved with transportation for mothers not staying at the hospital, wrappers to hold the baby, furniture/beds where mothers could conduct kangaroo mother care, rooms where mothers could spend the night with the baby,^24^^,^^48^ private spaces and dedicated resources.^40^^,^^106^

Without uniform knowledge and protocols within a facility, health-care workers were uncomfortable promoting kangaroo mother care.^16^^,^^27^^,^^42^^,^^99^^,^^107^ In-service training^82^^,^^100^ of health-care workers enhanced kangaroo mother care implementation.^56^ Virtual communication and training, often within facilities, allowed more nurses to be trained in kangaroo mother care despite busy schedules and staffing shortages.^36^ Expanding training to other health-care personnel, such as administrators and interns, also enabled care. Many nurses reported that integration of kangaroo mother care into pre-service and training curricula was beneficial.^36^^,^^57^

#### Context

Sociocultural context and sociocultural constructs of gender and roles of parents in childcare, men in the household and other family members influenced uptake.^79^^,^^85^^,^^96^ Parental and familial adherence to traditional newborn practices was reported as a barrier to kangaroo mother care.^105^ Traditional practices of early bathing and wrapping infants soon after birth were ingrained behaviours in many cultures that were difficult to change, even after training.^16^^,^^58^ In areas in which carrying the baby on the back was common, it seemed strange to place the baby on the front.^23^ In some contexts, it was considered unclean to have the mother carry the baby on her chest without a diaper.^79^

Please refer to the supplementary Table 4 (available at: http://www.who.int/volumes/94/2/15-157818) for full details of the included studies.

**Table 4:** Description of studies included in the systematic review on kangaroo mother care

Author, year	Country	Rural or urban	Study design	Sample size	Newborn characteristics	Kangaroo mother care components	Onset of skin-to-skin care	Provision of kangaroo mother care			Barriers and facilitators			
								Hours per day	Days		Caregivers	Health-care workers	Facilities	Policies and guidelines
Abul-Fadl, 2012^62^	Egypt	Mixed	Pop based surveillance, facility evaluation	1052 mothers	All ages	Skin-to-skin care	N/A	N/A	N/A		X^a^	X	X	–^a^
Aliganyira, 2014^29^	Uganda	Mixed	Facility evaluation, focus group/interview	11 facilities	N/A	Skin-to-skin care	N/A	N/A	N/A		–	X	X	–
Alves, 2007^84^	Brazil	Mixed	Chart review, focus group/ interview	33 dyads	Premature; N/A cut-off	N/A	Once eligible: N/A definition	N/A	N/A		X	–	–	–
de Araújo, 2010^33^	Brazil	Urban	Focus group/ interview	30 parents	Premature, ≥ 2000 g	N/A	Once eligible: N/A definition	5–6	N/A		X	X	X	–
Arivabene, 2010^28^	Brazil	Urban	Focus group/ interview	13 mothers	N/A	Skin-to-skin care	N/A	N/A	N/A		X	–	–	–
Bazzano, 2012^51^	Ghana	Rural	Focus group/ interview	9 mothers, 23 health-care workers	Low birthweight; N/A cut-off	Skin-to-skin care	N/A	N/A	N/A		X	–	–	–
Bergh, 2013^59^	Ghana	N/A	Facility evaluation	38 facilities	N/A	Skin-to-skin care, exclusive breastfeeding,	Immediately after birth	N/A	N/A		X	X	X	X
Bergh, 2003^100^	South Africa	Urban	Facility evaluation	2 facilities	N/A	N/A	N/A	N/A	N/A		–	X	X	–
Bergh, 2012^67^	Indonesia	Urban	Facility evaluation	10 facilities	N/A	N/A	N/A	N/A	N/A		X	–	–	–
Bergh, 2008^99^	South Africa	Mixed	Randomized controlled trial	36 facilities	N/A	N/A	N/A	N/A	N/A		X	X	X	–
Bergh, 2012^26^	Ghana	N/A	Pop based surveillance, facility evaluation	38 facilities	N/A	N/A	N/A	N/A	N/A		X	X	–	X
Bergh, 2009^83^	Ghana	N/A	Facility evaluation	4 regions (out of 10)	N/A	N/A	N/A	N/A	N/A		X	X	X	–
Bergh, 2012^25^	Malawi	N/A	Facility evaluation	14 facilities	N/A	N/A	N/A	N/A	N/A		X	X	X	X
Bergh, 2012^55^	Mali	N/A	Facility evaluation	7 facilities	N/A	Skin-to-skin care, exclusive breastfeeding, discharge, follow-up	N/A	N/A	N/A		X	X	X	X
Bergh, 2007^82^	Malawi	N/A	Facility evaluation	6 facilities	N/A	N/A	N/A	N/A	N/A		X	X	X	–
Bergh, 2012^47^	Rwanda	N/A	Facility evaluation	7 facilities	N/A	N/A	N/A	N/A	N/A		X	X	X	–
Bergh, 2012^24^	Uganda	N/A	Facility evaluation	11 facilities	N/A	N/A	N/A	N/A	N/A		X	X	X	X
Bergh, 2014^27^	Malawi, Mali, Rwanda, and Uganda	Urban	Facility evaluation, Focus group/interview	39 facilities	N/A	Skin-to-skin care	N/A	N/A	N/A		X	X	X	X
Blencowe, 2009^81^	Malawi	Urban	Prospective cohort	272 newborns	< 2000 g	N/A	Once eligible: N/A definition	N/A	N/A		X	–	X	–
Blencowe, 2005^80^	Malawi	Urban	Facility evaluation	1 facility	< 2000 g	Skin-to-skin care, exclusive breastfeeding, discharge, follow-up	N/A	N/A	N/A		X	–	–	–
Blomqvist, 2013^48^	Sweden	N/A	Focus group/ interview	76 mothers, 74 fathers	28–33 weeks, 740–2920 g	Skin-to-skin care	N/A	N/A	N/A		X	X	X	–
Blomqvist, 2011^39^	Sweden	Urban	Focus group/ interview	23 dyads	All ages	Skin-to-skin care, exclusive breastfeeding	N/A	N/A	N/A		X	X	X	–
Boo, 2007^105^	Malaysia	Urban	Randomized controlled trial	126 dyads	< 1501 g	Skin-to-skin care	Once eligible: N/A definition	1	10		X	X	X	–
Brimdyr, 2012^69^	Egypt	N/A	Focus group/ interview	40 nurses and health-care workers	N/A	Skin-to-skin care	Immediately after birth	1	1		X	X	X	–
Calais, 2010^49^	Sweden, Norway	Urban	Focus group/ interview	117 mothers, 107 fathers	Full term	Skin-to-skin care, discharge, follow-up	Immediately after birth	N/A	N/A		X	–	–	X
Castiblanco López, 2011^50^	Colombia	Urban	Focus group/ interview	8 mothers	< 36 weeks, 2320 g	N/A	N/A	N/A	N/A		X	–	X	–
Charpak, 2006^79^	15 developing countries	Mixed	Focus group/ interview	17 kangaroo mother care co-ordinators, 15 facilities	N/A	Skin-to-skin care, discharge, follow-up	Immediately after birth	N/A	N/A		X	X	X	–
Chia, 2006^111^	Australia	Urban	Focus group/ interview	34 nurses	N/A	Skin-to-skin care	N/A	N/A	N/A		X	X	X	–
Chisenga, 2015^11^	Malawi	Urban	Focus group/ interview	113 mothers	N/A	N/A	N/A	N/A	N/A		X	–	–	–
Colameo, 2006^85^	Brazil	Mixed	Cross sectional	28 facilities	Low birthweight; N/A cut-off	N/A	Once eligible: N/A definition	N/A	N/A		X	X	X	–
Cooper, 2014^73^	United States of America	Mixed	Pre-post	48 nurses and 101 parents	N/A	Skin-to-skin care	N/A	N/A	N/A		X	X	–	–
Crenshaw, 2012^71^	United States of America	N/A	Descriptive	261 dyads	Full term	Skin-to-skin care	≤ 2 mins after birth	N/A	1		X	X	X	–
Dalal, 2014^30^	India	Mixed	Cross sectional	145 HCPs	N/A	N/A	N/A	N/A	N/A		X	X	–	–
Dalbye, 2011^41^	Sweden, Norway	Urban	Focus group/ interview	20 mothers	Full term	Skin-to-skin care	Immediately after birth	N/A	N/A		X	X	–	–
Darmstadt, 2006^98^	India	Rural	Intervention	2063 mothers	All ages	Skin-to-skin care	N/A	N/A	N/A		X	–	X	–
De Vonderweid, 2003^104^	Italy	Mixed	Pop based surveillance	109 facilities	N/A	N/A	N/A	N/A	N/A		–	X	X	X
Duarte, 2001^97^	Brazil	Urban	Focus group/ interview	1 mother	Premature; N/A cut-off	Skin-to-skin care	N/A	N/A	38		X	–	X	–
Eichel, 2001^108^	United States of America	Urban	Facility evaluation	1 facility	N/A	N/A	N/A	N/A	N/A		X	X	X	X
Eleutério, 2008^114^	Brazil	Urban	Focus group/ interview	9 mothers	Premature; N/A cut-off	N/A	N/A	N/A	N/A		–	–	X	–
Engler, 2002^113^	United States of America	Mixed	Facility evaluation	537 facilities	N/A	N/A	N/A	N/A	N/A		X	X	X	–
Ferrarello, 2014^52^	United States of America	Urban	Focus group/ interview	15 mothers, 14 nurses	N/A	Skin-to-skin care	N/A	N/A	N/A		X	–	–	X
Flynn, 2010^66^	Ireland	Urban	Focus group/ interview	62 health-care workers	N/A	N/A	N/A	N/A	N/A		X	X	–	–
Freitas, 2007^86^	Brazil	N/A	Prospective cohort, descriptive	22 newborns	N/A	N/A	N/A	N/A	N/A		–	–	X	–
Furlan, 2003^87^	Brazil	Urban	Focus group/ interview	10 parents	Premature; N/A cut-off	Skin-to-skin care	Once eligible: N/A definition	10; mean	N/A		X	–	X	X
Gontijo, 2010^34^	Brazil	Mixed	Facility evaluation	293 facilities	N/A	Skin-to-skin care, exclusive breastfeeding	Once eligible: N/A definition	N/A	N/A		X	–	–	–
Gontijo, 2012^75^	Brazil	Mixed	Focus group/ interview	293 facilities	N/A	N/A	N/A	N/A	N/A		–	–	X	–
Gonya, 2013^63^	United States of America	Urban	Focus group/ interview	32 mothers	< 27 weeks	Skin-to-skin care	N/A	N/A	N/A		X	–	X	X
Haxton, 2012^36^	United States of America	Urban	Intervention, qualitative	30 mothers	All ages	Skin-to-skin care, exclusive breastfeeding	Within one hour after birth	3	1		X	X	X	X
Heinemann, 2013^40^	Sweden	N/A	Focus group/ interview	7 mothers, 6 fathers	< 27 weeks	Skin-to-skin care	N/A	N/A	N/A		X	–	X	–
Hendricks-Muñoz, 2010^44^	United States of America	Urban	Focus group/ interview	59 nurses	N/A	Skin-to-skin care	N/A	N/A	N/A		–	X	–	–
Hendricks-Muñoz, 2013^65^	United States of America	Urban	Focus group/ interview	143 mothers, 42 health-care workers	< 34 weeks	N/A	N/A	N/A	N/A		X	X	–	–
Hendricks-Muñoz, 2014^56^	United States of America	Urban	Prospective cohort	30 nurses	N/A	Skin-to-skin care	N/A	N/A	N/A		X	–	–	–
Hennig, 2006^88^	Brazil	Mixed	Cross sectional	148 doctors and nurses, 11 facilities	Low birthweight; N/A cut-off	N/A	Clinical stable	N/A	N/A		X	X	X	–
Higman, 2015^13^	England	Urban	Focus group/ interview	6 nurses and 51 clinicians	N/A	N/A	N/A	N/A	N/A		X	X	–	–
Hill, 2010^58^	Ghana	Mixed	Focus group/ interview	635 mothers, 14 villages	All ages	Skin-to-skin care	N/A	N/A	N/A		X	X	–	–
Hunter, 2014^32^	Bangladesh	Rural	Focus group/ interview	121 participants	N/A	N/A	N/A	N/A	N/A		X	X	–	–
Ibe, 2004^92^	Nigeria	Urban	Crossover	13 newborns, 11 mothers and female relatives	1200–1999 g	Skin-to-skin care	After enrolment	12	N/A		X	–	–	–
Johnson, 2007^106^	United States of America	Peri-urban/slum	Focus group/ interview	17 nurses	N/A	N/A	N/A	N/A	N/A		X	X	X	–
Johnston, 2011^37^	Canada	N/A	Randomized controlled trial crossover	62 newborns	28–36 weeks	Skin-to-skin care	≥ 15 minute before heel lance	≤ 1	2		X	–	–	–
Kambarami, 2002^96^	Zimbabwe	Urban	Focus group/ interview	N/A mothers	Low birthweight: N/A cut-off	N/A	N/A	N/A	N/A		X	–	X	–
Keshavarz, 2010^61^	Islamic Republic of Iran	Urban	Randomized controlled trial	160 dyads	Full term	Skin-to-skin care	2 hours after caesarean	3	N/A		X	–	–	–
Kostandy, 2008^112^	United States of America	N/A	Randomized controlled trial crossover	10 newborns	30–32 weeks	Skin-to-skin care	30 minute before heel stick	0.83	1		–	X	–	–
Kymre, 2013^45^	Sweden, Norway, Denmark	N/A	Focus group/ interview	18 nurses	N/A	Skin-to-skin care	N/A	N/A	N/A		X	X	–	–
Lee, 2012^42^	United States of America	Mixed	Focus group/ interview	69 health-care providers, 11 facilities	N/A	Skin-to-skin care	N/A	N/A	N/A		X	X	X	–
Legault, 1995^120^	Canada	Urban	Randomized controlled trial, pre-post, crossover	61 dyads	Premature: N/A cut-off 1000–1800 g	Skin-to-skin care	Once eligible: N/A definition	0.5	1		X	–	–	–
Lemmen, 2013^64^	Sweden	N/A	Focus group/ interview	12 families	24–35 weeks	Skin-to-skin care	N/A	N/A	N/A		X	X	–	–
Leonard, 2008^102^	South Africa	Urban	Focus group/ interview	6 parents	Premature: N/A cut-off	N/A	N/A	N/A	N/A		X	–	–	–
Lincetto, 1998^116^	Mozambique	Urban	Prospective cohort	246 newborns	< 2000 g	Skin-to-skin care, exclusive breastfeeding, discharge, follow-up	Stabilized health condition, presence of a sucking reflex, thermoregulation, mother's condition enabling her to care for the low birthweight infant, cessation of the infant's need for IV therapy, oxygen, photo-therapy or feeding by NG tube	> 20	N/A		X	X	X	–
Maastrup, 2012^53^	Denmark	N/A	Facility evaluation	19 facilities	N/A	Skin-to-skin care	18 out of 19 within 24 hour postpartum for stable preterm infant	N/A	N/A		X	–	–	–
Mallet, 2007^107^	France	N/A	Focus group/ interview	121 doctors and paramedical staff	N/A	N/A	N/A	N/A	N/A		X	X	X	–
Martins, 2008^115^	Brazil	Urban	Focus group/ interview	5 mothers	N/A	N/A	N/A	N/A	N/A		X	–	–	–
McMaster, 2000^78^	Papua New Guinea	Urban	Chart review, facility evaluation	109 newborns	< 1500 g	Skin-to-skin care	N/A	N/A	N/A		X	–	–	–
Moreira, 2009^101^	Brazil	Urban	Focus group/ interview	8 mothers	30–32 weeks, < 2000 g	Skin-to-skin care	Once eligible: N/A definition	N/A	N/A		X	–	–	–
Mörelius, 2015^15^	Sweden	Urban	Survey	129 nurses	All newborns	N/A	N/A	N/A	N/A		X	X	–	–
Mörelius, 2012^70^	Sweden	Mixed	Pop based surveillance	520 newborns	< 27 weeks	Skin-to-skin care	N/A	N/A	N/A		–	X	–	–
Nahidi, 2014^46^	Islamic Republic of Iran	Urban	Questionnaire	292 midwives	N/A	N/A	N/A	N/A	N/A		X	X	–	–
Namazzi, 2015^10^	Uganda	Rural	Randomized controlled trial	20 health facilities	All newborns	Skin-to-skin care	N/A	N/A	N/A		X	X	X	–
Neu, 1999^117^	N/A	Urban	Focus group/ interview	8 mothers, 1 father	Premature; N/A cut-off	Skin-to-skin care	N/A	1	2		X	X	X	–
Nguah, 2011^23^	Ghana	Urban	Prospective cohort	195 dyads	1000–2000 g	Skin-to-skin care, exclusive breastfeeding, follow-up	After admission in hospital and if mother was willing	N/A	N/A		X	–	–	–
Niela–Vilén, 2013^38^	Finland	Urban	Prospective cohort, qualitative	170 mothers, 381 staff	All NICU newborns	N/A	Immediately after birth	N/A	N/A		X	X	–	–
Nimbalkar, 2014^22^	India	Urban	Questionnaire	52 paediatricians	N/A	N/A	N/A	N/A	N/A		–	X	–	–
Nyqvist, 2008^95^	Sweden	N/A	Focus group/ interview	13 mothers	< 32 weeks	Skin-to-skin care, discharge, follow-up	N/A	N/A	N/A		X	X	X	X
Parmar, 2009^109^	India	Urban	Retrospective cohort	135 newborns	26–37 weeks, 550–2500 g	Skin-to-skin care	N/A	N/A	N/A		X	X	X	–
Pattinson, 2005^110^	South Africa	Mixed	Randomized controlled trial	34 facilities	N/A	N/A	N/A	N/A	N/A		–	–	X	–
Priya, 2004^93^	India	N/A	Crossover	30 dyads	Low birthweight; N/A cut-off	Skin-to-skin care	After routine care was observed and data were collected	2	2		X	–	–	–
Quasem, 2003^77^	Bangladesh	Urban	Focus group/ interview	35 mothers	All ages	Skin-to-skin care	N/A	N/A	N/A		X	–	X	–
Ramanathan 2001^103^	India	N/A	Randomized controlled trial	28 newborns	< 1500 g	N/A	Once eligible: N/A definition	≥ 4	N/A		X	X	–	–
Roller, 2005^94^	United States of America	N/A	Focus group/ interview	10 mothers	32–37 weeks	Skin-to-skin care	N/A	N/A	N/A		X	X	X	X
Sá, 2010^35^	Brazil	Urban	Focus group/ interview	10 mothers, 7 health-care providers	Premature; N/A cut-off	N/A	N/A	N/A	N/A		X	–	–	–
Sacks, 2013^21^	Honduras	Rural	Focus group/ interview	48–72 traditional birthing attendant (6 focus groups with 8–12 participants per group)	N/A	N/A	N/A	N/A	N/A		X	X	–	–
Santos, 2013^72^	Brazil	Urban	Focus group/ interview	12 mothers	Premature, low birthweight; N/A cut-off	Skin-to-skin care	N/A	N/A	N/A		X	–	X	–
Shamba, 2014^20^	United Republic of Tanzania	Mixed	Focus group/ interview	57 mothers and 14 traditional birthing attendants	N/A	N/A	N/A	N/A	N/A		X	–	–	–
Silva, 2014^74^	Brazil	Urban	Focus group/ interview	20 nursing technicians	N/A	N/A	N/A	N/A	N/A		X	X	–	–
Silva, 2015^12^	Brazil	Urban	Focus group/ interview	8 nurses	N/A	N/A	N/A	N/A	N/A		–	X	–	–
Silva, 2008^89^	Brazil	Urban	Focus group/ interview	5 dyads	Premature: N/A cut-off, < 1000–1550 g	Skin-to-skin care	Once eligible: N/A definition	≤ 24	Depended on mothers length of stay		X	–	–	–
Singh, 2012^19^	India	Mixed	Case control	145 662 newborns, 810 204 mothers	All ages	N/A	N/A	N/A	N/A		–	–	–	X
Sinha, 2014^18^	India	Rural	Focus group/ interview	320 mothers, 61 accredited social health activists, 19 home visits	N/A	Skin-to-skin care, exclusive breastfeeding	N/A	N/A	N/A		X	X	–	–
Sloan, 2008^76^	Bangladesh	Rural	Cluster randomized controlled trial	39 888 mothers	All ages	Skin-to-skin care	N/A	N/A	2; data available for first 2 days of life		X	X	–	–
Solomons, 2012^17^	South Africa	Urban	Cross sectional	30 mothers, 15 nurses	< 2500 g	N/A	N/A	N/A	N/A		X	X	X	–
Stikes, 2013^43^	United States of America	Urban	Focus group/ interview	56 nurses	N/A	Skin-to-skin care	N/A	N/A	N/A		X	X	X	X
Strand, 2014^54^	Sweden	N/A	Facility evaluation	126 staff	N/A	N/A	N/A	N/A	N/A		X	X	X	–
Tessier, 1998^119^	Colombia	Urban	Randomized controlled trial	488 newborns	< 2001 g	Skin-to-skin care, discharge, follow-up	Adapted to extra-uterine life and able to breastfeed	N/A	N/A		X	–	–	–
Toma, 2003^90^	Brazil	Urban	Focus group/ interview	14 mothers, 7 fathers	Premature: N/A cut-off, 1150–2300 g	N/A	Ranged from 3 to 39 days of life	N/A	N/A		X	–	–	–
Toma, 2007^91^	Brazil	Urban	Focus group/ interview	41 mothers	< 2000 g	N/A	Mean 18 days of life	N/A	N/A		X	–	X	–
Undefined author: Save the Children, 2011^57^	Ethiopia, Malawi, Mali, Mozambique, Nigeria, United Republic of Tanzania, Uganda, Bolivia, Indonesia, Nepal, Viet Nam	N/A	Facility evaluation	12 countries	N/A	N/A	N/A	N/A	N/A		X	X	X	X
Vesel, 2013^68^	Ghana	Rural	Cluster randomized controlled trial	98 zones	All ages	Skin-to-skin care	N/A	N/A	N/A		X	–	–	–
Wahlberg, 1992^118^	Sweden	Urban	Retrospective cohort	66 dyads	Premature; N/A cut-off	Skin-to-skin care	N/A	N/A	N/A		–	X	X	–
Waiswa, 2010^16^	Uganda	Rural	Focus group/ interview	30 health-care workers and mothers, 16 facilities	Premature; N/A cut-off	N/A	N/A	N/A	N/A		X	X	X	–
Waiswa, 2015^9^	Uganda	Rural	Cluster randomized controlled trial	395 women	All newborns	Skin-to-skin care, exclusive breastfeeding	N/A	N/A	N/A		X	–	–	–
Wobil, 2010^60^	Ghana	Urban	Facility evaluation	2 facilities	N/A	N/A	N/A	N/A	N/A		X	–	X	–
Zhang, 2014^31^	Singapore	Urban	Facility evaluation	1 ICU	Less than 34 weeks; Less than 1500 g	Skin-to-skin care	Once eligible: stable preterm or low birthweight babies, excluding infants with poor respiratory status, invasive lines, or parents who are depressed, not willing to do kangaroo mother care, having infectious skin disease on chest, unfit physically, or with flu-like symptoms.	At least 1 hour several times per day	N/A		X	X	X	–
Zwedberg, 2015^14^	Sweden	Urban	Focus group/ interview	8 midwives	N/A	N/A	N/A	N/A	N/A		X	X	–	–

^a ‘^X means included in the study and ‘–’ means not included in the study.

ICU: intensive care unit; N/A: not available; NICU: neonatal intensive care unit.

## Discussion

The core components of kangaroo mother care are skin-to-skin contact and feeding support. Additional features such as the frequency and location of early-discharge and follow-up depend on the context.^57^^,^^98^ Multiple factors influence the uptake of kangaroo mother care. To support the implementation of kangaroo mother care, context-specific materials such as guidelines, behaviour change materials, training curriculums, and job aids are needed. Simple interventions are more likely to be generalizable to a range of different contexts.^5^ When designing kangaroo mother care interventions, contextual factors and sociocultural norms need to be taken into account.

The stresses and stigma associated with having a preterm infant can hinder buy-in and support from parents and families for practicing kangaroo mother care. This problem is compounded by a lack of knowledge about kangaroo mother care among parents, families and health-care workers. Clear articulation of the benefits of kangaroo mother care for mothers and for newborns, creation of a community among parents, caregivers and health-care workers and engagement of fathers in childcare can help overcome these barriers. Collaboration among health-care workers, with shared goals and team commitments, partnering inexperienced nurses with nurses experienced in kangaroo mother care can also help.^42^^,^^106^^,^^108^

There are substantial barriers to kangaroo mother care within health systems, especially financing and service delivery. Dedicated financing for kangaroo mother care is critical for it to be seriously considered and implemented. Funding should consider creation of suitable environments (beds, wraps, chairs and private spaces), reducing burden of transport costs to mothers, home visits by community health workers and training parents to perform kangaroo mother care as independently as possible. Financing should be augmented with policies, guidelines, role definitions (to enable health-care workers to allocate protected time for kangaroo mother care), education (in service and pre-service) and monitoring systems that are suitably tailored for different settings (including in the community).

Logistic issues, such as time for travel and kangaroo mother care, can be challenging but could be partly overcome by incorporating targeted assistance and support and extension of visiting times. Buy-in from policy-makers is critical to promote kangaroo mother care, especially through policies like maternity and paternity leave.^42^^,^^107^ At the national level, kangaroo mother care should be integrated with essential newborn, maternal and child health guidelines, with appropriate monitoring and evaluation.^57^

We may not have captured all the programmatic reports and data available. In particular, most of the studies included in our review were published from regions with low neonatal mortality. This limits the generalizability of our findings.

## Conclusion

Prolonged skin-to-skin care demands time and energy from mothers recovering from labour and carers who may have other obligations. Many women are not aware of kangaroo mother care; health workers have not been trained or, if trained, do not promote such care. Kangaroo mother care may not be socially acceptable or even conflict with traditional customs. There is lack of standardization on who should receive kangaroo mother care and the presence of admissions criteria in neonatal units.

Kangaroo mother care should be practiced more systematically and consistently to enhance adoption^25^ and to build trust, with motivated trained staff, education of staff and parents, clear eligibility criteria, improved referral practices and creation of communities among kangaroo mother care participants through support groups. By addressing barriers and by building trust, effective uptake of kangaroo mother care into the health system will increase and this will help to improve neonatal survival. 

## 

KMC: kangaroo mother care.
